# Insecticide Susceptibility of *Phlebotomus argentipes* in Visceral Leishmaniasis Endemic Districts in India and Nepal

**DOI:** 10.1371/journal.pntd.0000859

**Published:** 2010-10-26

**Authors:** Diwakar Singh Dinesh, Murari Lal Das, Albert Picado, Lalita Roy, Suman Rijal, Shri Prakash Singh, Pradeep Das, Marleen Boelaert, Marc Coosemans

**Affiliations:** 1 Rajendra Memorial Research Institute of Medical Sciences, Patna, India; 2 B.P. Koirala Institute of Health Sciences, Dharan, Nepal; 3 Institute of Tropical Medicine Antwerp, Antwerp, Belgium; 4 London School of Hygiene and Tropical Medicine, London, United Kingdom; 5 Banaras Hindu University, Varanasi, India; National Institutes of Health, United States of America

## Abstract

**Objectives:**

To investigate the DDT and deltamethrin susceptibility of *Phlebotomus argentipes*, the vector of *Leishmania donovani*, responsible for visceral leishmaniasis (VL), in two countries (India and Nepal) with different histories of insecticide exposure.

**Methods:**

Standard WHO testing procedures were applied using 4% DDT and 0.05% deltamethrin impregnated papers. The effect of the physiological status (fed and unfed) of females on the outcome of the bioassays was assessed and the optimal time of exposure for deltamethrin was evaluated on a colony population. Field populations from both countries were tested.

**Results:**

Fed and unfed females responded in a similar way. For exposure time on field samples 60 min was adopted for both DDT and deltamethrin. In Bihar, knockdown and mortality with DDT was respectively 20 and 43%. In Nepal almost all sand flies were killed, except at the border with Bihar (mortality 62%). With 0.05% deltamethrin, between 96 and 100% of the sand flies were killed in both regions.

**Conclusions:**

Based on literature and present data 4% DDT and 0.05% deltamethrin seem to be acceptable discriminating concentrations to separate resistant from susceptible populations. Resistance to DDT was confirmed in Bihar and in a border village of Nepal, but the sand flies were still susceptible in villages more inside Nepal where only synthetic pyrethroids are used for indoor spraying. The low effectiveness of indoor spraying with DDT in Bihar to control VL can be partially explained by this resistance hence other classes of insecticides should be tested. In both countries *P. argentipes* sand flies were susceptible to deltamethrin.

## Author Summary

Visceral leishmaniasis (VL), also know as kala azar, is one of the major public health concerns India, Nepal and Bangladesh. In the Indian subcontinent, VL is caused by *Leishmania donovani* which is transmitted by *Phlebotomus argentipes*. To date, Indoor Residual Spraying (IRS) campaigns have been unable to control the disease. Vector resistance to the insecticides used has been postulated as one of the possible reasons explaining this failure. A number of studies in the region have shown a variable degree of resistance to DDT in areas where this insecticide has been widely used for IRS (mainly India). However there is no coordinated and standardized program to monitor resistance to insecticides in the region. In this study we tested *P. argentipes* susceptibility to DDT and deltamethrin in VL endemic villages in India and Nepal. The results confirmed the DDT resistance in India and in a border village of Nepal. *P. argentipes* from both countries were in general susceptible to deltamethrin, an insecticide used in some long lasting insecticidal nets.

## Introduction

Approximately 200 million people are at risk of visceral leishmaniasis (VL) – also known as kala-azar – in Bangladesh, India and Nepal [1]. In South East Asia, VL is caused by *Leishmania donovani* Laveran & Mesnil (Kinetoplastida: Trypanosomatidae) which is transmitted by *Phlebotomus argentipes* Annandale & Brunneti (Diptera: Psychodidae), the only incriminated vector in the region [2]. VL is fatal if untreated and current control measures rely on diagnosis and treatment of cases and Indoor Residual Spraying (IRS) to reduce or interrupt transmission in the affected communities. In India, two annual rounds of DDT spraying at 1 mg/m^2^ have been conducted in VL endemic districts since more than two decades [3]. In Nepal, the use of DDT to control VL was stopped in 1995 and IRS has been based since on synthetic pyrethroids (i.e alphacypermethrin or lambdacyhalothrin) targeting communities reporting at least one VL case in the previous year [4]. In Bangladesh vector-control activities are practically inexistent [5]. The use of Long Lasting Insecticidal Nets (LN), deltamethrin, alphacypermethrin or permethrin based [6], have been postulated as an alternative or complimentary approach as the current vector control strategies are failing to control VL in the region [7], [8]. Among other reasons, *P. argentipes* resistance to the insecticides used in the national programs may explain the lack of effect observed, particularly in India and Nepal. In a recent review, Ostyn et al. [8] reviewed the published reports on *P. argentipes* susceptibility to different insecticides in the Indian subcontinent since 1978. The results of this review show that DDT resistance has been reported in India since early 1990's but the results were variable and patchy. *P. argentipes* were consistently susceptible to DDT in Nepal and Bangladesh but the number of reports from those two countries was limited. Studies in the region showed susceptibility to deltamethrin, except for a report from Pondicherry, India [9]. However the methodologies used in those surveys were not standardized (i.e. insecticide concentration and time of exposure varied) and none of the studies applied the same protocol in different regions simultaneously.

In this paper we present the results of two studies on *P. argentipes* susceptibility to insecticides. First, a laboratory test to asses the influence of the physiological status of the sand fly on insecticide efficacy and to standardize the time of exposure to deltamethrin for field assays. Secondly, a field study was carried out to assess *P. argentipes* resistance to DDT and deltamethrin in VL endemic villages in India and Nepal.

## Materials and Methods

### Ethics statement

The protocol study was approved by the ethical review boards from the London School of Hygiene and Tropical Medicine, University of Antwerp, Rajendra Memorial Research Institute and B.P. Koirala Institute of Health Sciences. Written informed consent was obtained from the head of the household where the sand flies were collected.

### Study design

#### *P. argentipes* colony

Standard WHO testing procedures were applied to assess the insecticide resistance using the test-kit tubes [10]. First, *P. argentipes* sand flies from a colony kept at Rajendra Memorial Research Institute of Medical Sciences (RMRI - Patna, India) were used to assess the influence of the physiological status on susceptibility to insecticides and the expression of resistance. DDT was used at the concentration of 4% with an exposure time of 60 min. These exposure conditions were often used in the past and have shown to separate susceptible from resistant phenotypic populations of sand flies. For pyrethroids, limited results are available for discriminating both categories and 3 exposure times were tested (15, 30 and 60 min). Deltamethrin was used at the concentration of 0.05%, the discriminative dosage used for malaria vectors for an exposure of one hour. Based on these preliminary results from the colony population, susceptibility tests were then performed on wild *P. argentipes* populations. It is worth noting that the sand fly colony used in this study is periodically replenished with new wild-caught specimens and is not considered a reference strain.

#### Sand fly collection

**Figure 1:**
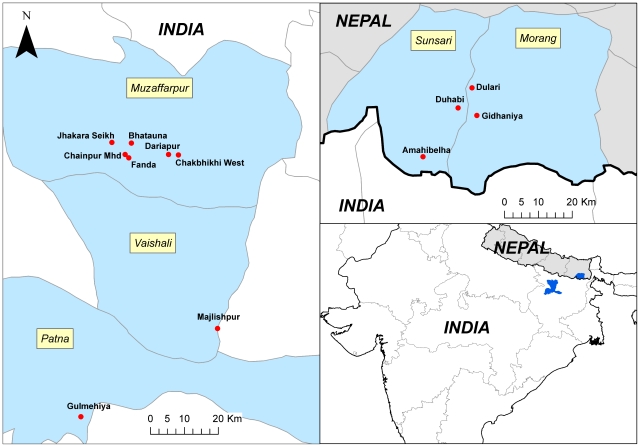
Map showing the location of the villages and districts where the susceptibility studies were conducted (in April-May 2008 and April-September 2009).

Field samples were collected in the early morning (from 6 to 8 a.m.) inside the houses by torchlight, with mouth aspirators in eight villages in Bihar, India and four villages in South-Eastern Nepal in April-May 2008 and April-September 2009. Those villages were selected as they were endemic for VL as reported in previous studies [11], [12], [13], [14]. The names and location of the study villages are provided in Fig. 1. About 20 houses were visited in each village for a one morning collection. In India, all bioassays were performed from April to May 2009. In Nepal, sand flies were tested for DDT from April-May 2008 and for deltamethrin from April-September 2009. Considering the limited density of sand flies all collected females regardless of their physiological status were tested with the tube tests. Control and insecticide-impregnated papers were supplied by the Vector Control Research Unit, Universiti Sains Malaysia (WHO collaborative Center) and were not used more than five times. Temperature (26–27°C) and relative humidity (45–85%) were noted during the tests. Several replicates (in Bihar 16 for DDT and 8 for deltamethrin; in Nepal 5 to 6 per study site for DDT and 6 to 9 per study site for deltamethrin), each of about 20 sand flies, according to the availability of the sand flies, were performed. Sand flies knockdown (KD) at the end of the exposure period were counted. Sand flies were then transferred to the holding tube supplied with 10% sugar solution and the mortality was recorded after 24 h. For each batch a control test was performed using the corresponding control papers (impregnated with Risella oil for DDT and Silicone Oil for deltamethrin). Mortality was corrected using Abbott's formula ([(% test mortality - % control mortality)×100]/[100 - % control mortality]) for mortalities in the control group between 5 and 20% [10]. Replicates from different villages in the same country were pooled together if no differences were observed in knockdown and mortality. Mean corrected knock down and mortality rates were calculated. Standard errors, representing the variation between replicates were also estimated.

## Results

### Colony population

**Figure 2:**
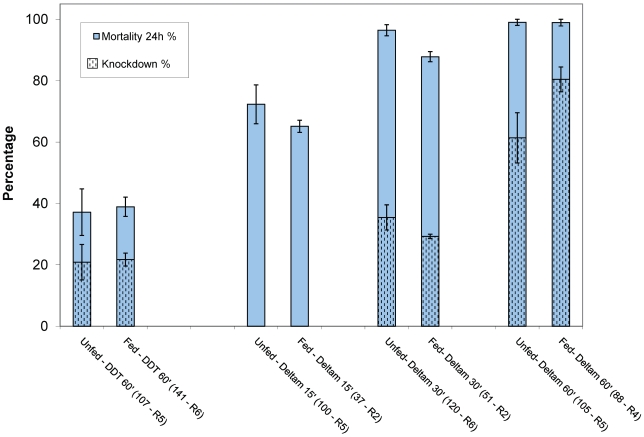
Susceptibility tests using the WHO bioassays with the colony population *P. argentipes* (Patna). E: unfed; F: fed. For deltamethrin (Deltam) 3 exposure periods have been tested (15, 30, 60 min); for DDT 60 min was used. The total number of specimens tested and the number of replicates (R) are shown in brackets. Bars represent the standard error of the mean.

No significant differences were observed between unfed (E) and fed (F) sand flies for knockdown and mortality (Fig. 2) for DDT (Chi Square KD: p = 0.46; mortality: p = 0.99) and for deltamethrin (Chi Square: KD: p = 0.17; mortality: p = 0.12). For DDT only 38% mortality (N = 248; 11 replicates) was observed indicating DDT resistance in this colony population. For deltamethrin knockdown increased with time of exposure and mortality after 60 min exposure reached 99% (N = 193; 9 replicates). The exposure time of 60 min with a concentration of 0.05% was further adopted for testing the field populations.

### Field populations

**Figure 3:**
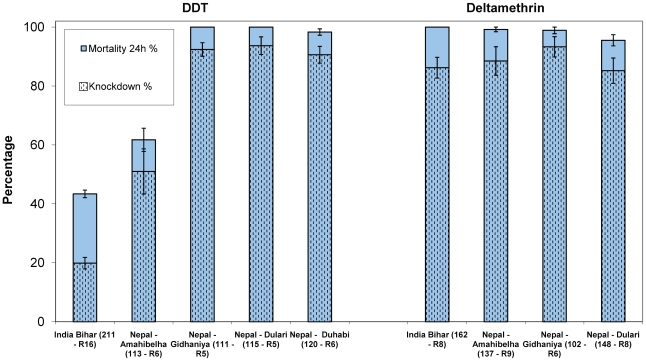
Susceptibility tests using the WHO bioassays on wild collected *P. argentipes* (Bihar and Nepal). For 4% DDT, 0.05% deltamethrin: 60 min exposure time. The total number of specimens tested and the number of replicates (R) are shown in brackets. For Bihar all replicates performed on specimens coming from the eight study villages are pooled together. Bars represent the standard error of the mean.

For Bihar all replicates performed on specimens coming from the eight study villages were put together as no difference occurred in knockdown and mortality among the study sites. For DDT, knockdown was of 20% and only 43% died after 24 h (1 h exposure, N = 211; 16 replicates) suggesting DDT resistance. Deltamethrin 0.05% induced a knockdown of 86% and a mortality of 100% (N = 162; 8 replicates) (Fig. 3).

In Nepal, the results were presented by village as there were differences between study sites. DDT resistance was only observed in one of the villages (i.e. Amahibelha) (KD 51%, mortality 62%, N = 113; 6 replicates) while full susceptibility was observed in the other three sites. For deltamethrin, knockdown fluctuated between 85 and 93% and mortality between 96–99% (Fig. 3). Mean mortalities and knockdown rates were very similar to the rates calculated on total specimens tested.

Results of the bioassays are provided in detail as Dataset S1.

## Discussion

No discriminating concentrations or time to kill all susceptible specimens have been established for sand flies as is the case for malaria vectors [10]. Based on literature data [8], [15] 4% DDT and 1 h exposure seems to be an acceptable discriminating concentration. The sand fly colony of Patna can then be considered as resistant to DDT, as well as the wild population in the study area of Bihar. This DDT resistance in the colony of *P. argentipes* in RMRI is not surprising as it is regularly mixed with wild specimens and cannot be considered a reference strain. Previous data (1998–1999) in the area [16] showed a patchy distribution of DDT resistance (mortality between 100 and 71%). The observed mortality of around 40% in present study could suggest an increasing trend in DDT resistance in Bihar. However, dose or time response assays are needed to compare the levels of resistance between the different populations [17]. In Nepal, DDT resistance was only observed in the study site of Amahibelha (mortality 62%), a location close to the border with Bihar (Fig. 1), while *P. argentipes* was susceptible in the other 3 more inland located study sites. In Nepal the use of DDT for IRS was stopped in early 1990's and from 1995 the IRS policy was mainly based on the use of pyrethroids (mainly alphacypermethrin) but only in villages with VL cases [4]. This underlines once more that DDT resistance in *P. argentipes* has been mainly attributed to indoor spraying with this insecticide and its frequency of application [8], [18], but the use of sublethal doses as consequence of poor management and supervision of the IRS control programs may also enhance the selective pressure.

As no fully susceptible reference strain of *P. argentipes* was available, it was not possible to estimate a discriminating concentration with deltamethrin. Deltamethrin 0.05% is the discriminating concentration established for anopheline vectors, but it is not obvious to extrapolate this to sand flies or *P. argentipes*. In Brazil, bioassays with 0.05% deltamethrin were used and a clear difference between the insecticide susceptibility of two sand fly populations was observed [17]. In that study the sand fly population without previous specific insecticide exposure, a Lethal Time 50% (LT50) of 25 min was obtained and all sand flies died after one 1 h. In the population exposed to sand fly control measures using pyrethroids, LT50 was significantly higher (40 min) and the mortality was only 62% after 1 h [17]. Bioassays performed on the colony population of RMRI indicate a LT50 lower (<15 min) than the one observed in the most susceptible population in Brazil. One hour exposure induced a knockdown of around 70% and a mortality of 99% and these exposure conditions were further maintained for testing field populations. Similar results were obtained for the field populations (KD: 81–92%; mortality 95–100%) suggesting, and contrasting with the Brazilian study, a relatively good susceptibility to deltamethrin of the wild *P. argentipes* populations of Nepal and Bihar. Moreover, in *P. argentipes*, there is no indication of DDT-deltamethrin cross resistance, commonly found in anophelines where target Kdr resistance is present [19]. So far only metabolic mechanisms have been reported in sand flies [15], [17] and acetylcholinesterase and esterase-based insecticide resistance mechanisms have been observed in *P. argentipes* of Sri Lanka which probably arose from IRS with Malathion of the Anti-Malaria Campaign [20].

The limited but significant reduction (25%) of *P. argentipes* densities induced by mass use of deltamethrin-based long lasting insecticidal nets (LNs) observed in a trial conducted recently in the same areas in India and Nepal [21] cannot therefore be explained by a low susceptibility to deltamethrin but resides in the behavior of vector. Indeed *P. argentipes*, although known as being endophilic, are mainly opportunistic blood feeders and feed in a significant proportion on bovines[22]. Hence, this will reduce the mass effect of LNs on *P. argentipes* populations. The current failure to control the transmission of *L. donovani* in the region relying on IRS with DDT can be partially explained by the resistance to this compound and other insecticides should be evaluated to replace it. However, the first requirement for a successful control program remains the quality of IRS implementation.

## Supporting Information

Dataset S1 Results of the bioassay tests.
(0.18 MB XLS)
